# Round the Hospitals

**Published:** 1916-04-22

**Authors:** 


### ROUND THE HOSPITALS.

In a paper read by Miss Adelaide Nutting at the Pan-American Congress held in Washington in January of this year, the author gave us a very interesting view of nursing conditions in America. Miss Nutting mentions that in many hospitals the quarters and living conditions for the pupils are inadequate and inappropriate, whilst in all of them the hours of work are painfully long. On the other hand, Miss Nutting tells of noteworthy improvements in the direction of uniformity of standards; the lengthening to three years of the period of training; marked improvement in methods of instruction; and the acquired subjects are much more carefully and thoroughly taught than they were some years ago. Miss Nutting points to progress made in a more rigid exclusion of unsuitable candidates for admission to the training school. There is no doubt that the careful selection of nurses is one of the most important means of promoting the welfare of the nursing profession. The practice of admitting for training undisciplined and wilfully careless or incapable pupil-nurses cannot fail to act detrimentally by lowering the standard both of work in the wards and of the estimation in which nurses are held by the general public.

At a special meeting of the General Council of the Royal British Nurses' Association held on March 27, though acknowledging that the effect of the College of Nursing on the Association would be to cause loss of membership and serious financial loss, Mr. Comyns Berkeley moved "That the Royal British Nurses' Association support the foundation of a College of Nursing and accept representation on its Council." Mr. Comyns Berkeley stated that he thought the College would be a great success, and expressed his belief that the R.B.N.A. would certainly be able to help the College by the large experience the Association possesses of nursing matters. He stated that he would like in time to come to feel that the Association had a part in the founding of the College of Nursing.

Since July 1915 members of the Women's Emergency Corps have been on duty at the Middlesex Hospital, where they take the place of porters who have left to join the Army. These women, who work in bands of five, act as trolley workers, and convey the patients to and from the wards and theatres. They have won golden opinions from the medical staff, and both the matron and secretary have expressed appreciation of the services they have rendered. Miss Sylvester, hon. secretary of the Corps, informs us that she will be glad to supply trolley workers to other hospitals requiring assistance in this conveyance of patients. This has proved an excellent way of solving the problem arising from a shortage of porters, and we believe our readers will be glad to hear of Miss Sylvester's offer. The address of the Corps is York Place, Baker Street, W.

The Cape Medical Council, according to the *South African Nursing Record*, has once more rejected the proposal to establish a second-grade certificate for coloured nurses, so the problem of how to provide adequate skilled nursing for coloured patients remains unsolved. All will agree that the native population should receive efficient care in sickness, but there is no doubt that white nurses should not be employed for this purpose. At present, though coloured nurses are eligible, the standard of education required is such that only exceptional native women are able to obtain a certificate of registration. There is apparently a growing demand for coloured nurses, and some hospitals are employing them to a considerable extent not as ward-maids or orderlies, but for actual nursing duties. Native women nurses will be employed by the European as well as the native population when they have had two or more years' experience in such hospitals. The *South African Nursing Record* condemns the action of the Cape Medical Council, for it shows that it can only result in lowering the standard of nursing in the Colony. The demand for coloured nurses is pressing, and the status of native nurses must be faced and determined without delay.

Tripe is generally regarded as an extremely common and vulgar dish. Yet, carefully prepared, stewed tripe is a most palatable, easily digested, and nutritious food. It contains protein, fat, and a certain amount of carbohydrate in a most assimilable form. It can be given to convalescents beyond the milk stage with entire confidence when they have no insuperable preconceived prejudices against it. At the present time, when fish and fowls are scarce and dear, "Tripe Diet" could in many cases be substituted for "Fish Diet" and "Chicken Diet" one or two days a week in hospitals and in general practice. Its only drawback is the care necessary in its preparation. The tripe must be absolutely fresh, most carefully cleansed and "dressed," and be submitted to prolonged, slow, and careful cooking. When the stew is noticeably darker than milk, and the "meat" less tender than spring chicken, it is faulty.

